# From lab design to clinical translation: photoacoustic endoscopy

**DOI:** 10.1016/j.pacs.2026.100876

**Published:** 2026-08-25

**Authors:** Qingrong Xia, Xiaojing Gong

**Affiliations:** a Affiliated Nanhua Hospital, University of South China, Hengyang, Hunan 421001, China; b Research Center for Biomedical Optics and Molecular Imaging, Key Laboratory of Biomedical Imaging Science and System, Shenzhen Institute of Advanced Technology, Chinese Academy of Sciences, Shenzhen, Guangdong 518055, China; c Guangdong Provincial Key Laboratory of Biomedical Optical Imaging, Shenzhen Key Laboratory for Molecular Imaging, Shenzhen, Guangdong 518055, China

**Keywords:** Photoacoustic endoscopy, Optical excitation, Acoustic detection, Scanning mechanism, Multimodal endoscopy, Clinical translation

## Abstract

Photoacoustic endoscopy (PAE) integrates pulsed optical excitation and acoustic detection within miniaturized probes for intraluminal and intracavitary imaging. By combining optical absorption contrast with ultrasound-based detection, PAE complements conventional endoscopy by visualizing subsurface vasculature and providing functional or compositional contrast, including hemoglobin oxygenation, lipid-rich tissue, collagen-associated signals, and molecular probes. This review summarizes scenario-driven PAE development from engineering design to translational application, focusing on optical excitation, acoustic detection, and scanning strategies. We analyze trade-offs among resolution, depth of field, sensitivity, imaging speed, field of view, probe dimensions, and workflow compatibility, and review representative applications in cardiovascular, gastrointestinal, reproductive, urinary, respiratory, and surgical-guidance settings. Current evidence remains largely preclinical or early feasibility. Future translation requires standardized quantitative metrics, laser safety evaluation, robust engineering and prospective clinical validation.

## 1 Introduction

Accurate assessment of diseases in deep luminal and cavity organs remains challenging because existing imaging techniques often cannot simultaneously provide sufficient penetration depth, spatial resolution, and functional sensitivity. Endoscopic imaging brings the probe closer to the lesion and improves local visualization. Conventional white-light endoscopy mainly provides surface morphology. Endoscopic ultrasound (EUS) offers limited spatial resolution, optical coherence tomography (OCT) is restricted by shallow penetration, and fluorescence imaging (FI) often lacks depth-resolved capability. Therefore, there remains a strong need for endoscopic techniques that can provide depth-resolved structural, functional, and molecular information within confined luminal or cavity environments [1], [2], [3], [4], [5].

Photoacoustic endoscopy (PAE) combines optical absorption contrast with ultrasonic detection, thereby bridging the functional sensitivity of optical imaging with the depth-resolved capability of ultrasound. By selecting appropriate excitation wavelengths and system architectures, PAE can visualize microvascular networks, estimate hemoglobin-related parameters, map lipid- or collagen-associated PA contrast, and detect signals from exogenous molecular probes. In addition, PAE can be integrated with EUS, OCT, and FI to provide complementary structure-function-molecule information [6], [7], [8], [9], [10], [11], [12]. These features make PAE a promising platform for disease characterization in cardiovascular, gastrointestinal (GI), reproductive, urinary, respiratory, and surgical-guidance scenarios.

**Fig. 1:**
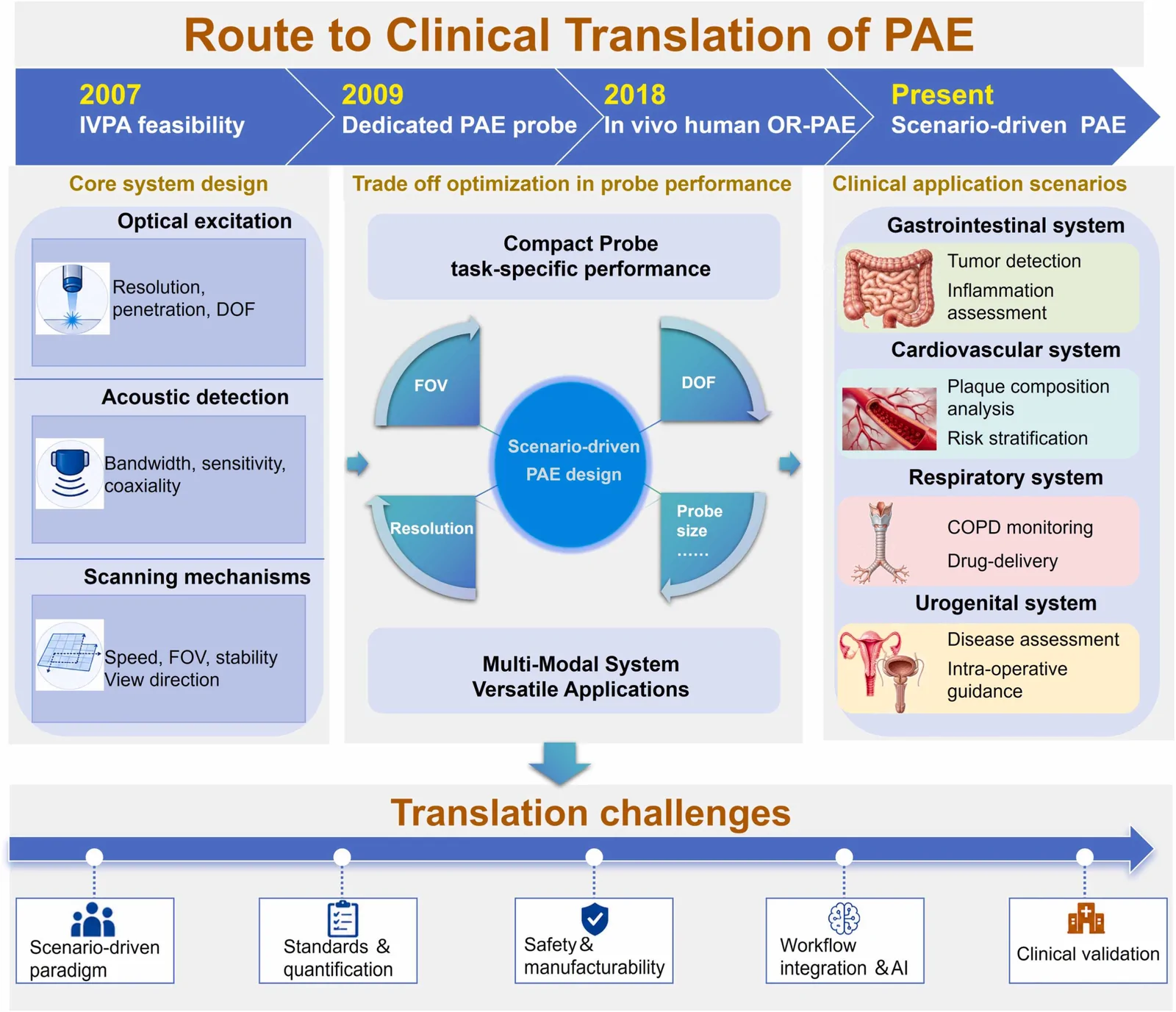
PAE system design and clinical translation in intraluminal imaging. IVPA, intravascular photoacoustic imaging; OR, optical-resolution; PAE, photoacoustic endoscopy; COPD, Chronic obstructive pulmonary disease; DOF, depth of field; FOV, field of view; AI, artificial intelligence. Notes: Some illustrative elements in this figure were generated using ChatGPT and were further arranged and edited by the authors.

However, the main barrier to clinical translation is not a single component, but the coupling of multiple performance requirements under strict probe-size constraints. Optical excitation must balance wavelength selection, fluence, focal-spot size, depth of field (DOF), and energy delivery. Acoustic detection must balance bandwidth, sensitivity, coaxial integration, and probe diameter. Scanning mechanisms must balance field of view (FOV), imaging speed, motion stability, and workflow compatibility. These constraints explain why no universal PAE architecture is suitable for all organs; instead, system design must be matched to specific anatomical environments and clinical tasks. This review is organized around the concept that clinical demand drives PAE system evolution **(**Fig. 1**)**. Table 1 is organized primarily along the ‘translational stage’. It selects several translational milestones in the development of PAE and pairs each with the enabling technologies that made them possible. This correspondence is intended to highlight which core technical advances have played pivotal roles in moving PAE from conceptual demonstration toward clinical translation.

**Table 1:** Representative work in the PAE translational route. IVPA, intravascular photoacoustic imaging; IVUS, intravascular ultrasound; PAE, photoacoustic endoscopy; EUS, endoscopic ultrasound; GI, gastrointestinal; OCT, optical coherence tomography; OR, optical-resolution; PAEM, photoacoustic endomicroscopy; DSCC, dynamic-static decoupled coaxial core; 3D, three dimensional.

**Year**	**Representative work**	**Technology category**	**Evidence level/** **Translational stage**	**Key significance**
2007	S. Sethuraman, et al., S. Y. Emelianov*. IEEE TUFFC [13]	IVPA imaging using an IVUS catheter	IVPA Prototype validation	First proposal and validation of IVPA imaging technology.
2009	J.-M. Yang, et al., L.V. Wang*. Opt Lett [14]	Dedicated PAE probe architecture	PAE Prototype validation	Build a complete PAE probe, performed circumferential scanning, and achieved lumen inside imaging.
2011	K. Jansen, et al., G. van Soest*. Opt Lett [8]	IVPA for coronary plaque imaging	Ex vivo human tissue validation	Ex vivo human coronary plaque imaging and lipid-sensitive IVPA validation.
2012	J.-M. Yang, et al., L.V. Wang*. Nat Med [9]	Multimodal PAE-EUS	In vivo animal validation	First in vivo GI tract PAE.
2015	J.-M. Yang, et al., L.V. Wang*. Biomed Opt Express [10]	OR-PAE	In vivo animal validation	Enabled microvascular imaging with OR PAE.
2018	Y. Qu, et al., L.V. Wang*. J Biomed Opt [15]	Transvaginal PAE	In vivo human feasibility	First in vivo human transvaginal for cervical microvascular imaging.
2019	H. He, et al., V. Ntziachristos*. J Biophotonics [16]	Capsule-based PAE	Preclinical prototype validation	Demonstrated capsule-compatible optoacoustic endoscopic imaging.
2022	M. Kim, et al., J.-M. Yang*. Photoacoustics [17]	Endoscope-compatible PAE-EUS mini-probe	In vivo large-animal validation	PAE-EUS mini-probe compatible with standard GI endoscope channels.
2022	Y. Liang, et al., B.-O. Guan*. Nat Commun [18]	Optical heterodyne detection-based functional GI PAE	In vivo small-animal validation	Optical heterodyne detection enabled high-sensitivity functional GI PAE.
2024	Q. Xia, et al., X. Gong*. Photoacoustics [19]	Intrauterine PAE	In vivo small-animal validation	First uterine PAE imaging in vivo, achieving endometrial microvascular assessment.
2025	X. Liang, et al., L. Xi*. Photoacoustics [20]	Multi-scenario PAE	Multi-model in vivo human/small animal validation	Cavity-adaptive PAE across in vivo environments, including human oral, rabbit cervical, rat rectal.
2025	T. Zhao, et al., W. Xia*. Photoacoustics [21]	Ultrathin multimode-fiber PAEM	Ultrathin probe ex vivo validation	Ultrathin multimode-fiber PAEM for faster high-resolution minimally invasive imaging.
2026	X. Wen, et al., X. Ji*. Photonics Res [22]	Dynamic perfusion PAE	In vivo preclinical validation	Ultrafast DSCC catheter for 3D intracavitary blood-perfusion imaging.
2026	Q. Li, et al., V. Ntziachristos*. Nat Biomed Eng [23]	Tethered PAE-OCT capsule	Ex vivo human-tissue validation	Human Barrett’s esophageal neoplasia assessment.

## 2 Engineering trade-offs in PAE

PAE performance is primarily determined by optical excitation, acoustic detection, and scanning mechanism. Optical excitation defines contrast and spatial scale; acoustic detection determines sensitivity and integration geometry; and scanning controls FOV, speed, and stability. Therefore, PAE design should balance multiple coupled metrics rather than maximize a single parameter.

### 2.1 Optical excitation: contrast and spatial scale

Optical excitation determines the primary contrast source and spatial imaging scale in PAE, while also constraining resolution, DOF, and energy-delivery efficiency. Laser sources and molecular probes at different wavelengths broaden the applications of PAE, extending from vascular morphology to oxygenation status, lipid distribution, collagen/fibrosis assessment, and biomarker-oriented molecular information [18], [24], [25], [26], [27], [28], [29], [30], [31], [32], [33], [34], [35]. Thus, optical excitation design must balance focal-spot size, DOF, pulse-energy delivery, and probe miniaturization at different wavelengths. In cardiovascular imaging, near-infrared wavelengths are commonly used to enhance photoacoustic signals from lipid-rich plaques, stent structures, and related pathological components. These applications usually prioritize compositional identification in deeper tissue, high detection sensitivity, and signal stability in blood-containing environments. Therefore, weakly focused or acoustic-resolution imaging schemes are often adopted to achieve a larger DOF and higher pulse-energy delivery, although at the expense of relatively limited lateral resolution. In contrast, PAE of the GI tract, reproductive system, and other luminal or cavity organs mainly focuses on microvascular networks, perfusion, and oxygenation in mucosal or superficial tissues. These applications often use visible or near-infrared hemoglobin-sensitive wavelengths, relying on the absorption differences between oxyhemoglobin and deoxyhemoglobin to image microvascular morphology and oxygenation. For such superficial microcirculation imaging, a smaller optical spot and higher lateral resolution are usually required, but this inevitably reduces the DOF and deliverable pulse energy.

**Fig. 2:**
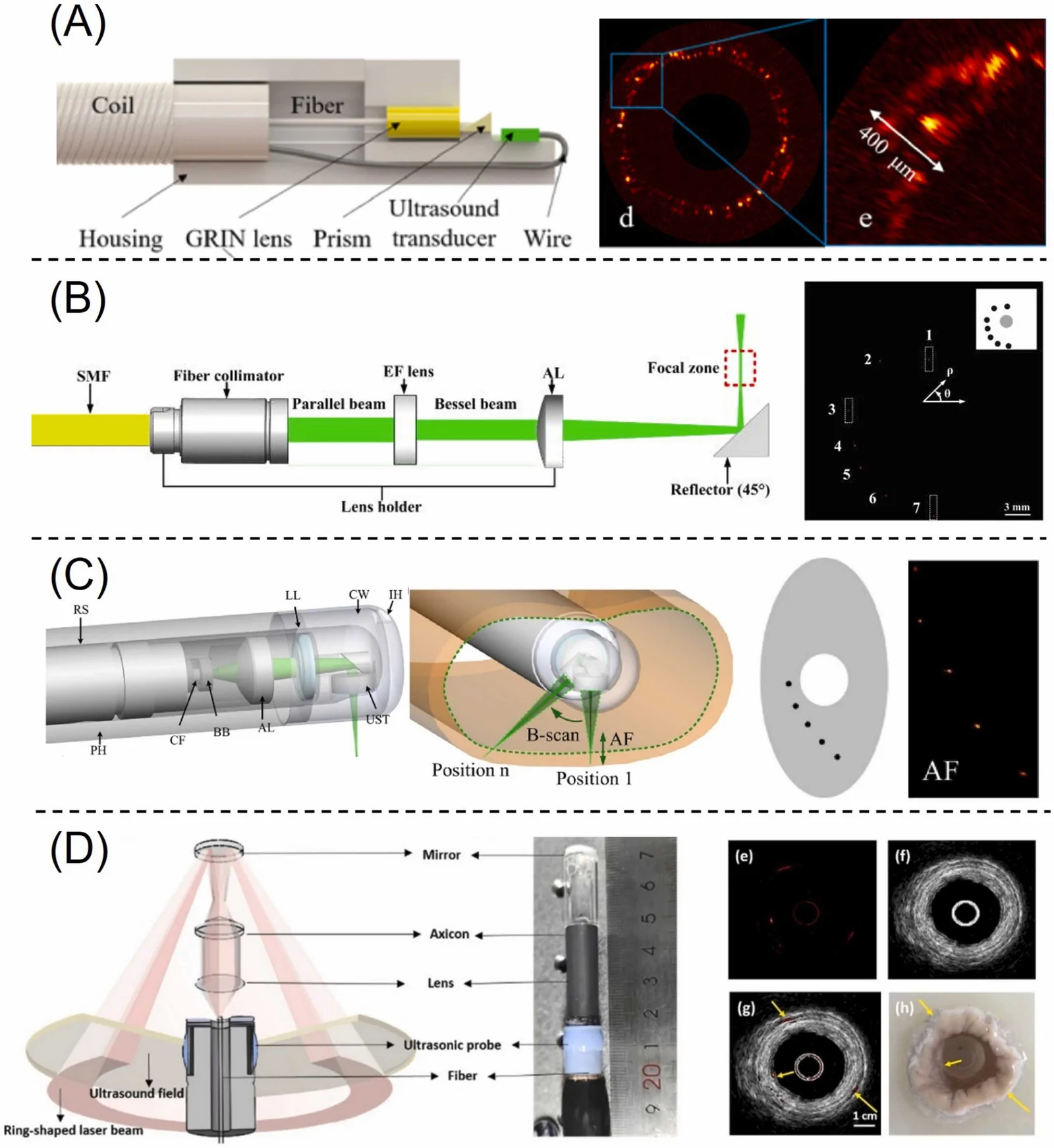
Representative PAE systems enabled by different optical excitation strategies. (A) Gaussian-beam-based PAE probe design and representative rectal imaging in an animal model [36]. (B) Optical configuration for Bessel-beam excitation and representative imaging of carbon fibers [38]. (C) Schematic illustration and tungsten-wire imaging performance of a liquid-lens-based PAE probe [39]. (D) Design, photograph, and representative rectal imaging of a PAE probe based on tunable annular illumination [42]. Figures reproduced with publisher's permission.

Gaussian-beam excitation remains the most widely used strategy in PAE because of its simple configuration, mature optical components, and ease of catheter integration. Weakly focused Gaussian beams are more suitable for acoustic-resolution imaging that requires greater penetration depth and sensitivity, such as intravascular lipid-rich plaque imaging. In contrast, tightly focused Gaussian beams provide smaller optical spots and are suitable for optical-resolution imaging of superficial microvascular networks in the GI and reproductive tracts [8], [10], [13], [15], [19], [36], [37]. Representative work on Gaussian-beam-based PAE probe design and rectal imaging in an animal model is shown in Fig. 2**A**. However, Gaussian beams are inherently limited by the trade-off between resolution and DOF: stronger focusing improves lateral resolution but shortens the effective DOF and restricts deliverable pulse energy and imaging depth.

To extend the effective imaging range, Bessel-beam excitation and tunable-focus strategies have been introduced into PAE [38], [39], [40]. Representative work on Bessel-beam excitation and imaging is shown in Fig. 2**B**. Bessel beams can maintain relatively stable lateral resolution over an extended axial range, making them suitable for intestinal imaging and other scenarios with uneven tissue surfaces or variable working distances [38]. Tunable-focus methods use liquid lenses (Fig. 2**C**) or optomechanical structures to dynamically adjust the focal plane, allowing the system to maintain favorable resolution at different imaging depths [39], [40]. Ring-shaped excitation is mainly used in array-based or ring-shaped acoustic detection systems. By matching the optical illumination with the acoustic field of a ring array, this strategy can reduce dependence on point-by-point mechanical scanning and improve cross-sectional imaging speed and FOV coverage [41], [42] (Fig. 2**D**). It is therefore more suitable for relatively large luminal organs, such as the rectum, or for applications requiring large-FOV and high-speed cross-sectional imaging.

In recent years, excitation strategies based on fiber properties and modal control have also attracted increasing attention. GRIN-fiber mode cleaning can improve beam quality and lipid-detection sensitivity in IVPA; high-fluence relay-based disposable photoacoustic-ultrasound endoscopy can enhance energy-delivery efficiency and clinical workflow compatibility; and lensed-fiber-based PAE further supports microvascular imaging in small luminal organs [43], [44], [45].

Overall, optical excitation in PAE is evolving from simple beam delivery toward scenario-oriented coordination of contrast, resolution, DOF, and energy distribution. The key is not to pursue the absolute superiority of a single beam format, but to select the most appropriate excitation strategy according to target-organ anatomy, imaging depth, functional information requirements, and catheter-size constraints. In addition, for functional and compositional PAE, excitation design should additionally include wavelength-specific pulse-energy normalization, calibration of fiber and optical-component transmission, and assessment of fluence-induced spectral distortion.

### 2.2 Acoustic detection: sensitivity and integration geometry

Acoustic detection determines the ability of PAE to receive photoacoustic signals and is a key factor affecting signal-to-noise ratio, imaging depth, and the visibility of deep targets. The center frequency and bandwidth of the transducer mainly determine axial resolution, while receiving sensitivity governs the detectability of weakly absorbing targets and deep signals [46]. Meanwhile, the device size directly affects the receiving aperture, sensitivity, optical-acoustic overlap, and catheter diameter. Therefore, acoustic detection design requires a balance among sensitivity, bandwidth, size, and optical-acoustic coupling efficiency. For targets located mainly in superficial tissues, such as mucosal or endometrial microvessels, the requirement for penetration depth is relatively limited; thus, high-frequency and small-sized detectors may be preferred to improve resolution [18], [19]. In contrast, imaging scenarios involving deeper structures, such as the gastric wall, intestinal wall, uterine wall, or vascular wall, rely more heavily on higher detection sensitivity and greater effective imaging depth [25], [26], [27], [28], [47], [48].

**Fig. 3:**
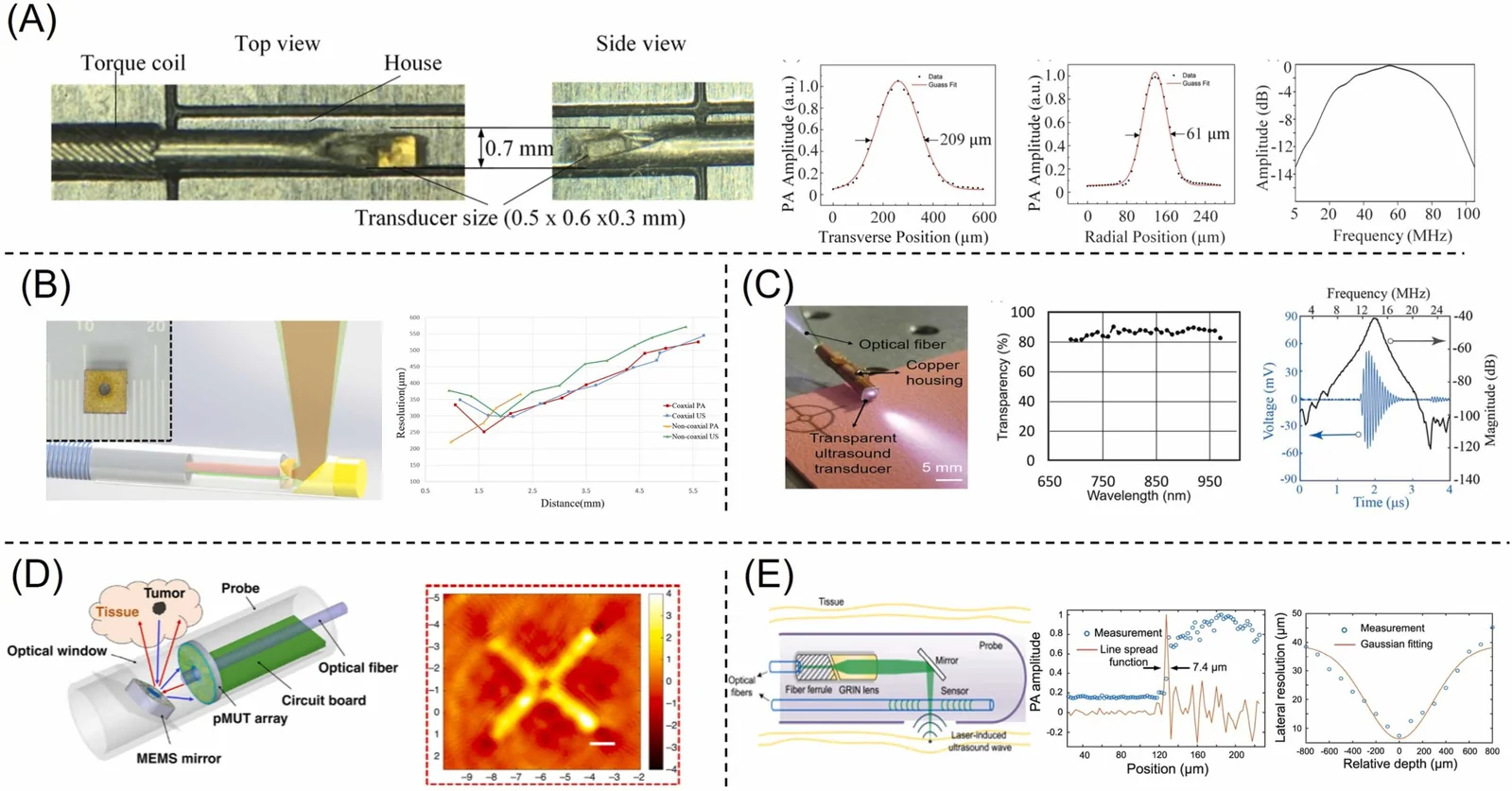
Overview of acoustic detection strategies in PAE. (A) Conventional piezoelectric transducer-based PAE probe and its spatial resolution characterization [51]. (B) Hollow-transducer-based PAE probe enabling coaxial optical–acoustic integration and its imaging range performance [62]. (C) Transparent-transducer-based PAE probe and corresponding ultrasound echo response characterization [63]. (D) Dual Frequency PMUT-array-based PAE probe and corresponding fused photoacoustic image [58]. (E) All-optical PAE probe based on optical heterodyne detection and its corresponding resolution characterization [18]. PMUT, piezoelectric micromachined ultrasonic transducer. Figures reproduced with publisher's permission.

Conventional bulk piezoelectric transducers remain the most mature and engineering-feasible solution owing to their stable performance, established fabrication process, and compatibility with pulse-echo ultrasound imaging. They have been widely used in early PAE systems for intravascular and gastrointestinal imaging, typically with a side-by-side optical–acoustic layout; a representative probe and its characterization is shown in Fig. 3**A** [13], [37], [49], [50], [51]. This configuration is simple and practical, but the optical excitation and acoustic detection fields only partially overlap, which reduces the effective detection region and signal-collection efficiency. In addition, bulk dicing and assembly processes limit further miniaturization and high-density arrays in endoscopic probes.

To address the miniaturization and array-integration bottlenecks of bulk piezoelectric devices, capacitive micromachined ultrasonic transducers (CMUTs) and piezoelectric micromachined ultrasonic transducers (PMUTs) provide a Microelectromechanical Systems (MEMS) -based route for compact PAE systems [52], [53]. Compared with conventional bulk transducers, CMUTs and PMUTs offer advantages in device miniaturization, multi-frequency, high-density array integration, wafer-level batch fabrication, and flexible aperture design. They can be configured as single element transducers, linear arrays, annular arrays, forward-viewing arrays, or multichannel detectors, making them versatile acoustic-detection platforms for compact PAE-EUS endoscopic systems [52], [53], [54], [55], [56], [57], [58], [59], [60]. However, their sensitivity, acoustic coupling, electrical interconnection, packaging, and long-term reliability under endoscopic conditions still require further improvement.

For the common problem of insufficient optical–acoustic overlap in non-coaxial detector configurations, hollow and transparent transducer architectures have been developed to improve coaxial integration. Hollow transducers represent a typical approach to achieving coaxiality in conventional transducers and are relatively straightforward to implement (Fig. 3**B**). They incorporate a central aperture for optical delivery, thereby enabling coaxial or near-coaxial alignment between optical excitation and acoustic detection [61], [62]. This design is useful for luminal imaging scenarios, such as rectal, esophageal, or intravascular PAE, where improved optical–acoustic coupling is required. However, the central aperture reduces the acoustic receiving area and may lower detection sensitivity. Benefiting from the design flexibility of MEMS fabrication, high-density or multi-frequency hollow arrays can be realized using CMUTs and PMUTs, which may partially compensate for aperture loss and improve array-level performance **(**Fig. 3**D**) [55], [56], [58]. Transparent transducers further improve coaxial integration by allowing the excitation beam to pass directly through the detector while preserving most of the effective acoustic receiving aperture (Fig. 3**C**) [63], [64]. This architecture is suitable for imaging scenarios requiring deeper detection, accurate structural localization, and dual- or multimodal integration. In addition to transparent piezoelectric transducers, transparent CMUTs have also been explored for photoacoustic applications, showing the feasibility of combining optical transmission with acoustic detection in a MEMS-based platform [65]. These studies suggest that transparent or optically accessible CMUT designs may provide another route toward compact coaxial PAE-EUS probes. Nevertheless, transparent detectors still face challenges in material selection, electrode transparency, matching-layer design, device consistency, packaging, and cost.

All-optical acoustic detection provides a new direction for further improving miniaturization, bandwidth, and sensitivity. Detectors based on Fabry–Pérot (FP) cavities, optical heterodyne detection (Fig. 3E), microring resonators (MRRs), and fiber Bragg gratings (FBGs) offer advantages such as small size, broad bandwidth, high sensitivity, and easy fiber-based integration [66], [67], [68], [69], [70], [71], [72], [73], [74]. These features make them attractive for ultraminiature forward-viewing probes, needle-based endoscopic probes, minimally invasive surgical guidance, and high-resolution experimental platforms. However, all-optical detection remains less clinically validated than conventional piezoelectric probes, and its integration with standard pulse-echo ultrasound imaging is less straightforward than that of piezoelectric transducers.

Overall, acoustic detection in PAE is evolving along two coupled directions: improving optical–acoustic coupling efficiency and advancing detector miniaturization and integration. Conventional piezoelectric transducers provide mature, stable detection; MEMS-based CMUTs and PMUTs offer miniaturized, arrayed device platforms that can be adapted to both off-axis and coaxial architectures; hollow or transparent transducers optimize coaxial optical–acoustic alignment; and all-optical sensors explore the limits of fiberized, broadband, and ultracompact detection. Future detector selection should be guided by specific application scenarios, with careful consideration of sensitivity, bandwidth, aperture, optical–acoustic overlap, probe size, and quantitative calibration.

### 2.3 Scanning mechanisms: FOV, speed, and stability

The scanning mechanism is one of the most distinctive technical features that differentiates PAE from external photoacoustic imaging. It determines how the system covers the luminal wall or cavity surface and directly affects the FOV, imaging speed, volumetric coverage, motion stability, and clinical workflow compatibility. Because PAE relies on miniature probes to access different anatomical spaces, the scanning design should not simply pursue higher speed or a larger FOV. Instead, it must balance scanning range, volumetric imaging speed, probe size, motion stability, and optical energy load according to specific application scenarios. For long tubular organs such as blood vessels, the esophagus, and the airway, complete circumferential coverage and high rotational stability are usually required. In contrast, for open or semi-open cavities such as the stomach, oral cavity, and uterine cavity, forward visualization, local target positioning, and interventional guidance are more important.

**Fig. 4:**
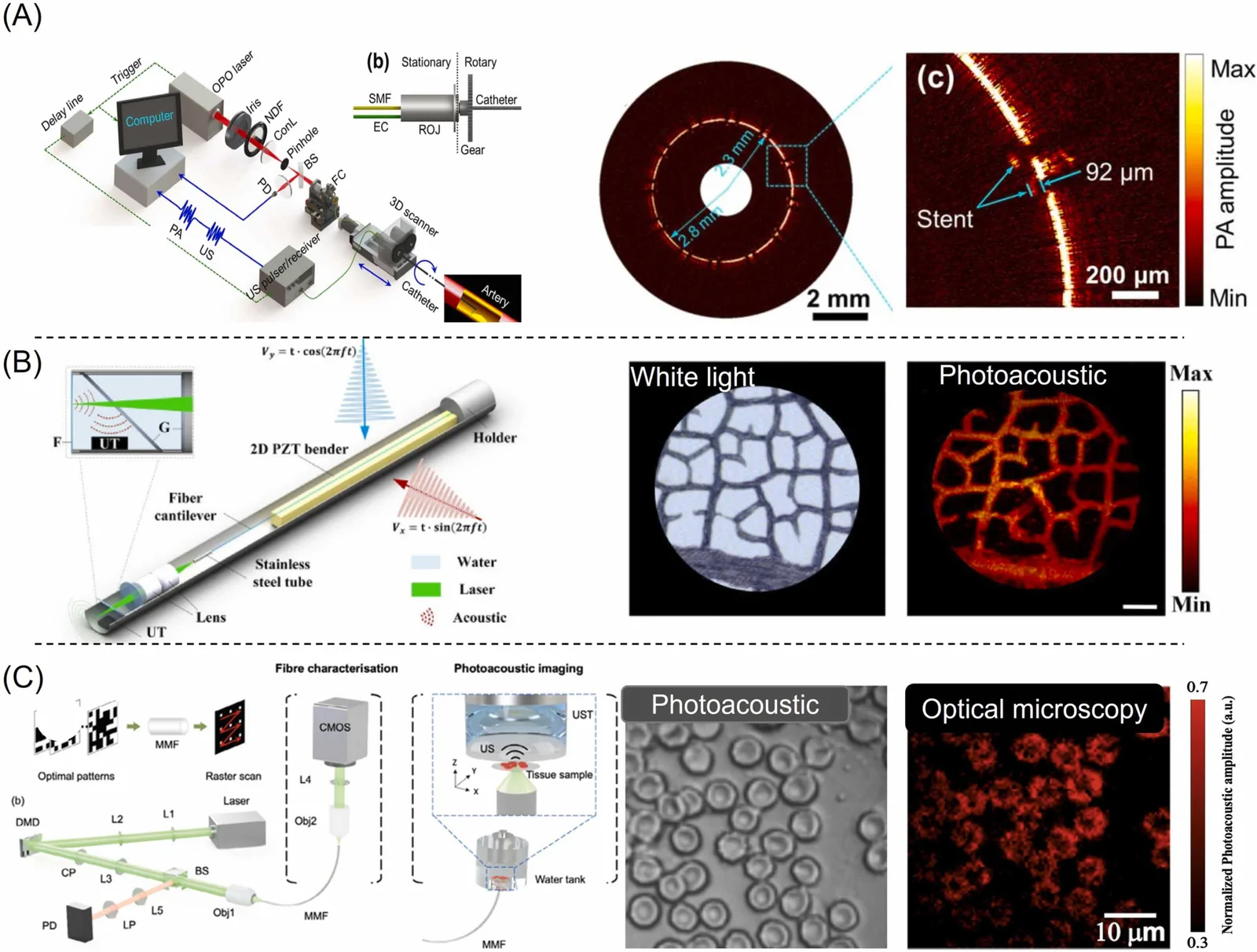
Representative PAE systems based on different scanning mechanisms. (A) Side-viewing PAE system with scanning illustration and representative imaging results [49]. (B) Forward-viewing PAE probe design and its imaging results compared with white light [78]. (C) Schematic of a wavefront-shaping-based PAE system and its imaging results compared with optical microscopy [83]. Figures reproduced with publisher's permission.

Side-viewing circumferential scanning is currently the most mature PAE scanning strategy for tubular organs; a representative work is shown in Fig. 4**A**. According to the driving position, side-viewing scanning can be divided into distal-end scanning and proximal-end scanning. In distal-end scanning, a micromotor placed at the probe tip directly drives rotation, providing better scanning stability and reducing non-uniform rotational distortion (NURD). However, this design increases the distal probe size and rigid length, making further miniaturization difficult. In proximal-end scanning, an external motor drives probe rotation through a torque coil, which helps reduce the distal probe size and improves flexibility for interventional access. Nevertheless, in curved luminal pathways, friction and non-uniform torque transmission can easily introduce NURD and other artifacts [8], [14], [49], [75], [76]. Therefore, side-viewing scanning is well suited for continuous imaging of long luminal organs, but further clinical translation requires improved rotational stability, synchronization accuracy, and motion-artifact correction.

Two-dimensional planar scanning can theoretically be used for both side-viewing and forward-viewing imaging, but it is currently more commonly applied in forward-viewing PAE. Forward-viewing scanning (Fig. 4**B**) shifts the imaging region from the lateral wall to the area ahead of the probe, making it more suitable for image guidance, lesion localization, and interventional procedures. It is particularly useful in scenarios that require direct visualization of the forward target area, such as the stomach. In recent years, MEMS mirrors, Lead Zirconate Titanate (PZT) scanners, and fiber scanners have been used in forward-viewing PAE, improving scanning speed and spatial coverage to some extent [71], [77], [78], [79]. However, these approaches still face challenges in FOV expansion, control of distal rigid length, packaging reliability, and uneven acoustic sensitivity across the two-dimensional imaging field. Therefore, they are more suitable for relatively large cavities or local high-resolution imaging of target regions, whereas their use in ultranarrow lumens remains limited by probe size and stability.

Wavefront-shaping-based multimode fiber scanning (Fig. 4**C**) provides a new route toward ultrathin, mechanical-scanning-free PAE. By controlling the incident wavefront of a multimode fiber (MMF), this approach generates a controllable focal spot or speckle pattern at the distal end, avoiding conventional lenses, mirrors, and mechanical rotation. This may reduce the distal probe size to the fiber scale. In recent years, multifocal excitation, compressed sensing, and deep-learning-based computation have further improved the scanning speed and computational efficiency of these systems [21], [80], [81], [82], [83], [84]. However, wavefront shaping strongly depends on the stability of the multimode-fiber transmission matrix or speckle field. It is highly sensitive to fiber bending, temperature changes, and environmental perturbations, and also requires complex calibration and computational algorithms. Therefore, this technique is currently more suitable as a frontier exploratory direction for ultrathin PAE and computational endoscopic imaging. Its long-term stability and engineering scalability in real in vivo environments still need further validation.

Recent advances in scanning technology increasingly emphasize the coordinated optimization of speed, FOV, and stability. Dual-scanning photoacoustic endomicroscopy (PAEM) combines wide-angle scanning with local spiral scanning, enabling both rapid large-area screening and high-quality local microvascular imaging [85]. Multi-scenario PAE adjusts imaging geometry to adapt a single platform to different anatomical environments [20]. Dynamic-static decoupled coaxial core (DSCC) design further improves high-speed rotational stability and enables ultrafast volumetric imaging of intracavitary blood perfusion [22]. These studies indicate that future PAE scanning mechanisms should not focus only on increasing scanning speed. Instead, it should improve motion correction, scanning synchronization, three-dimensional coverage efficiency, and workflow compatibility according to specific clinical scenarios. should not focus only on increasing speed, but also improve

Overall, current PAE scanning mechanisms are becoming increasingly diverse. No single strategy is superior in all performance metrics, and the optimal choice depends on the specific clinical application. Future scanning design should be selected according to the target anatomy and clinical task, balancing FOV, volumetric imaging speed, probe size, motion stability, and ease of clinical operation.

Table 2 is structured around ‘technical architecture and performance trade-offs’. In the optical-excitation dimension, the emphasis is on how successive technical iterations have progressively broken through the trade-off between lateral resolution and DOF under the strict constraint of probe diameter. In the acoustic-detection dimension, the focus is on achieving efficient photoacoustic coaxial integration within the limited probe volume while continuously increasing signal bandwidth to improve axial resolution. In the scanning-mechanism dimension, both classic distal- and proximal-driving schemes, as well as more recent innovations that substantially reduce probe size while markedly increasing imaging speed and FOV, are included. The overarching selection principle is that, for the most foundational and classic technical approaches, early, widely recognized seminal works are prioritized; for the remaining categories, preference was given to recent studies with relatively complete performance reporting and clear improvements in core parameters under relevant physical constraints.

**Table 2:** Representative PAE systems and engineering trade-offs. Primary and secondary trade-off parameters are defined differently for each technological category. IVUS, intravascular ultrasound; fps, frames per second; GI, gastrointestinal; DOF, depth of field; FOV, field of view; MMF, multimode fiber; FBG, fiber Bragg gratings; MEMS, microelectromechanical systems; OD, outer diameter; PAEM, photoacoustic endomicroscopy; N.A, not applicable or not reported.

**Core element**	**Category**	**Implementation**	**Primary Trade-off** **Parameters**			**Secondary Trade-off Parameters**		**Suitable scenario**	**Representative ref.**
			**Probe size (mm)**	**Lateral Resolution (μm)**	**DOF (mm)**	**Imaging speed**	**FOV**		
Optical excitation	Traditional Beam	Gaussian Beam	3.4 (OD)	9.2	∼ 0.72 (*Estimated*)^a^	4 fps	270°	GI microvascular imaging	2015, [10] L. Wang
	Special Beam	Bessel beam	8 (OD, with sheath) × 120 (Length)	41.8–62	9.48 (in water)	2 fps	360°	Large-DOF intestinal microvascular imaging	2019, [38] S. Yang
		Tunable focusing	2.9 (OD)	3–5	Tunable DOF 0.038 mm-0.118 mm in 3.2 mm range	0.029 fps (*Estimated*)^b^	4 mm (Raster scanning B-Scan Range)	High resolution imaging over extended DOF	2023, [40] S. Chen
		Ring-shaped illumination	11 (OD, with sheath)	544–1283	DOF: N.A Imaging distance: 12–30	100 fps	360°	Large-FOV intestinal assessment	2023, [42] X. Gong

**Core element**	**Category**	**Implementation**	**Primary Trade-off** **parameters**			**Secondary Trade-off parameters**		**Suitable scenario**	**Representative ref.**
			**Probe size (mm)**	**Axial** **Resolution (μm)**	**Central Frequency/ 6 dB Bandwidth**	**Imaging speed**	**FOV**		
Acoustic detection	Piezo-electric transducer	Conventional IVUS-compatible design	0.83 (only IVUS catheter)	38	40 MHz Bandwidth: 63%	0.08 fps (*Estimated*)c	360°	Vulnerable plaque assessment	2007, [13] S. Emelianov
	Coaxial integration of Piezoelectric transducer	Hollow piezoelectric transducer	12 (OD)	60	15 MHz Bandwidth: 150%	5 fps	240°(*Estimated*)^d^	Rectum vessel imaging	2018, [61] D. Xing
		Transparent ultrasound transducer	1.8 (OD)	∼70	21.2 MHz Bandwidth: 61%	10 fps	360°	GI wall and microvasculature	2024, [63] C. Kim
	Optical detection	FBG	0.35 (longer dimension of 2 fibers)	8.5	160 MHz (20% Peak)	0.12 fps (*Estimated*)^e^	6.5 mm × 7 mm (Raster scanning)	High- resolution microvascular imaging with a small probe	2024, [74] V. Ntziachristos

**Core element**	**Category**	**Implementation**	**Primary Trade-off** **parameters**			**Secondary Trade-off parameters**		**Suitable scenario**	**Representative ref.**
			**Probe size** **(mm)**	**Imaging speed**	**FOV**	**Resolution (μm)**	**Working** **Distance (mm)**		
Scanning mechanism	Side-viewing	Distal scanning	18.6 (OD, with sheath)	2.5 fps	360°	80 (Lateral)	6.8–10.5	Volumetric imaging of esophagus	2019 [16] V. Ntziachristos
		Proximal scanning	0.9 (OD)	100 fps	360°	< 200 (Lateral)	0.6–1.5	Atherosclerotic plaque assessment	2020, [76] X. Gong
	Forward-viewing	MEMS-based scanning	6.0 (OD)× 30 (Length)	50 fps (*Estimated*)^f^	0.4 mm × 0.4 mm	10.6 (Lateral)	*N.A.*	Wide-field GI imaging	2017, [77] L. Xi
		Fiber scanning	5 (OD)	0.5 fps	3 mm (Diameter)	15.6 (Lateral)	*N.A.*	Forward-view gastric vascular imaging	2023, [78] S. Yang
	Wavefront shaping	MMF-based scanning	0.14 (OD)	2–57 fps	0.05 mm × 0.05 mm	1.7–3 (Lateral)	0.1	Video-rate PAEM	2022, [83] W. Xia
	Adaptive scanning	Dual scanning	9 (OD) × 140 (Length)	1.25 fps (circular scanning); 0.25 fps (angular scanning)	2.5 mm (circular FOV); 2.5 mm × 27.5 mm (angular FOV)	12.4 (Lateral)	8 (Focus- adjustment range)	Dual-mode high-resolution GI microvascular imaging	2025, [85] L. Xi

a The DOF was estimated under the Gaussian-beam assumption (same as the reported): $\mathrm{DOF}=2\frac{\pi \omega_{0}^{2}}{{\lambda M}^{2}}$, where $\omega_{0}^{2}$ is 7.8 μm, λ is 532 nm, M^2^ is 1;

b The overall time for multi‑focus scanning was ∼35 s, the multi-focus B-Scan scanning speed is estimated as 1/35 = 0.029 fps;

c It was estimated that with 250 A-lines per B-scan (vessel-phantom imaging) and a 20-Hz laser repetition rate, the frame rate was 0.08 fps (i.e., 20/250 = 0.08 fps);

d It was estimated that the effective FOV = 360°– 120°(blocked) = 240°, where the 120° blockage was attributed to the opaque supporting bridge of the probe housing.

e It was estimated that with a 1 mm scan range at a 2 μm step, average 20 times per A-line, and a laser repetition rate of 1.2 kHz, the frame rate was 0.12 fps (i.e., 1200/((1 mm/2 μm) × 20) fps).

f It was estimated that acquiring 400 B-scans took approximately 8 s, corresponding to a frame rate of 50 fps (400 B-scans/8 s = 50 fps).

## 3 Scenario-driven PAE applications

PAE applications should be evaluated according to clinical need, anatomical constraint, information requirement, and workflow compatibility. Cardiovascular PAE emphasizes small-diameter, high-speed, and lipid-specific contrast; GI PAE emphasizes large-FOV functional and molecular assessment; and reproductive, urinary, respiratory, and surgical-guidance applications emphasize safe access and scenario-adapted probe design, highlighting the need for scenario-specific probe design. To date, most reported applications remain at the preclinical or early feasibility stage. Their future clinical relevance will depend on whether PAE-derived information is reproducible and provides indispensable value beyond established imaging modalities.

### 3.1 Cardiovascular PAE: plaque composition and multimodal assessment

Cardiovascular photoacoustic endoscopy, commonly referred to as intravascular photoacoustic imaging (IVPA), is primarily aimed at identifying vulnerable components of atherosclerotic plaques, including lipid-rich necrotic cores, inflammatory activity, neovascularization, and stent-related abnormalities. These clinical targets impose stringent requirements on IVPA, including high sensitivity for compositional detection, fast and stable imaging performance, catheter miniaturization, and strict laser safety control.

**Fig. 5:**
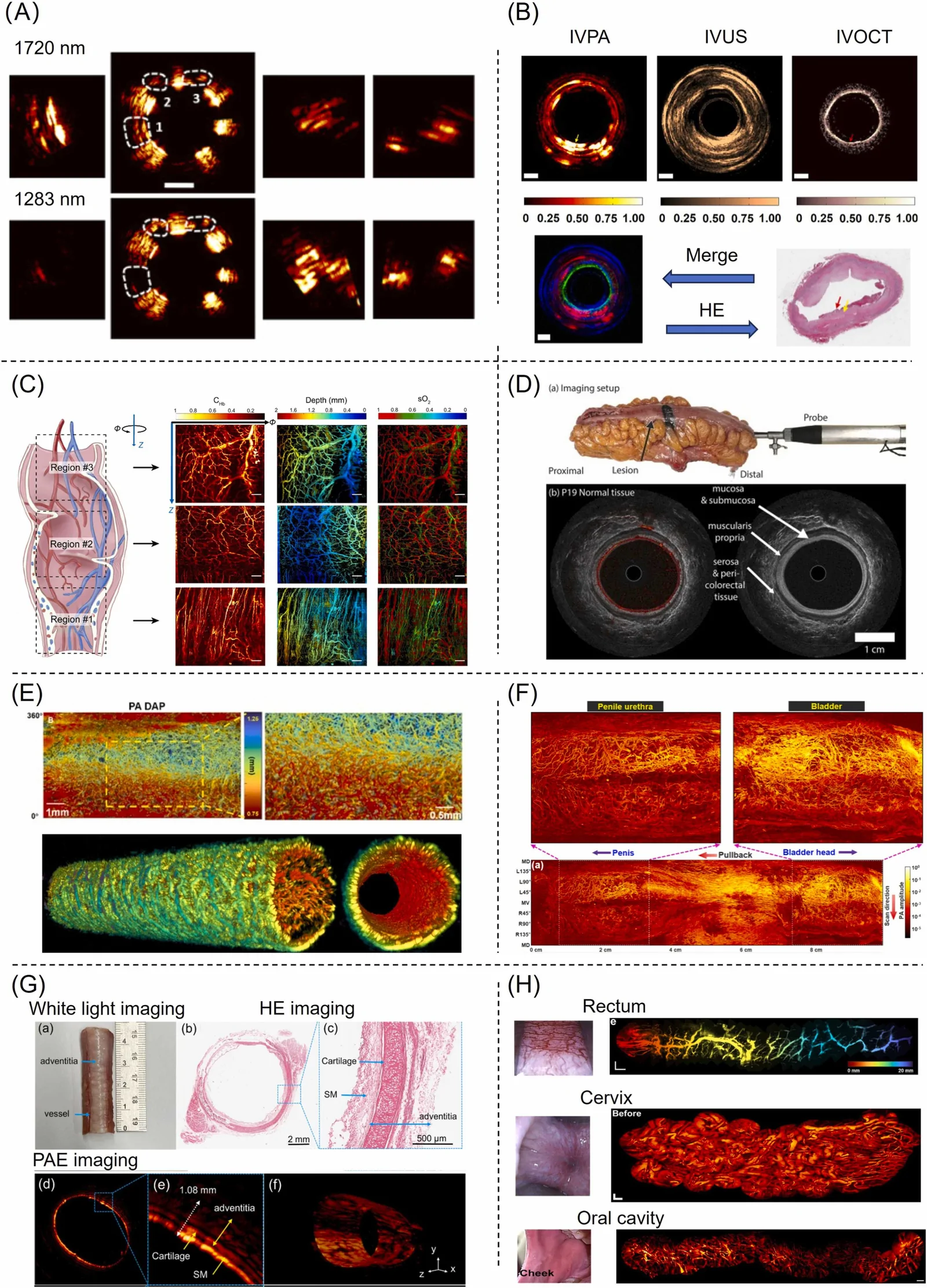
Applications of PAE in GI, cardiovascular, urogenital, respiratory systems and multi-scenario imaging, using single- and multimodal imaging strategies. (A) IVPA of lipid-mimicking phantoms at different wavelengths [28]. (B) Multimodal IVPA–IVUS–OCT imaging of ex vivo human atherosclerotic vessels with corresponding histopathological validation [89]. (C) Single-modality PAE imaging of the rectum in a small animal model [36]. (D) Dual-modality PAE–EUS imaging of ex vivo human rectal tissue [95]. (E) PAE of the reproductive system in a small animal model [19]. (F) PAE of the urinary system in a small animal model [103]. (G) PAE of the respiratory system in a small animal model, with comparisons to white-light imaging and histopathology [105]. (H) Multi-scenario PAE imaging results across different organ systems [20]. Figures reproduced with publisher's permission.

Early IVPA studies mainly demonstrated technical feasibility (Fig. 5**A**). Investigators first showed that photoacoustic imaging could be implemented using intravascular ultrasound (IVUS)-compatible catheters, followed by ex vivo validation in fresh human coronary atherosclerotic arteries and lipid-specific spectroscopic imaging [8], [13], [31], [32], [37]. Subsequent multispectral IVPA further improved the accuracy of lipid identification and characterization of intraplaque components [28], [29], [30], [31], [32], [33], while molecularly targeted probes enabled imaging of plaque inflammation and neovascularization [86], [87].

As the clinical demand has shifted from single-component lipid detection toward comprehensive plaque-risk assessment, IVPA has increasingly moved toward multimodal integration. Recent IVPA systems combining IVUS, OCT, and elastography have enabled simultaneous assessment of plaque morphology, lipid distribution, fibrous-cap structure, and mechanical properties, a representative work is shown in Fig. 5**B** [28], [88], [89]. These studies suggest that IVPA is more likely to achieve broad clinical use as an important component of a multimodal intravascular imaging platform.

For in vivo imaging and progress toward clinical translation, high-speed IVPA-IVUS systems have been developed to reduce motion artifacts, shorten acquisition time, and improve procedural feasibility [50], [76], [90], [91]. In parallel, studies on laser exposure and tissue safety have provided important evidence supporting the use of IVPA within appropriate safety limits [92], [93].

Overall, the potential of IVPA for plaque composition assessment has been extensively investigated, particularly for lipid-associated imaging and multimodal characterization of vascular structure. However, further large-scale prospective clinical studies are needed to confirm its incremental diagnostic and prognostic value beyond that of IVUS, OCT, or other modalities. Because cardiovascular disease affects a large patient population, IVPA has remained one of the most intensively investigated application areas in PAE. However, intravascular imaging is technically demanding because of the narrow vascular lumen, complex blood background, vascular pulsation, and strict requirements for probe size, imaging speed, and laser safety. Therefore, although IVPA has steadily advanced from ex vivo human-tissue validation and animal model studies toward human clinical application, its translation remains cautious and gradual. Further progress will require continued improvements in catheter reliability, blood-background suppression, real-time imaging, quantitative standardization, and safety validation.

### 3.2 Gastrointestinal PAE: from microvascular imaging to functional and molecular assessment

GI PAE is one of the most extensively investigated application areas in the field, with studies spanning catheter, balloon, capsule, and multimodal probe configurations. The development and progression of GI tumors, inflammation, and fibrosis are often accompanied by changes in vascular morphology, wall structure, oxygenation, collagen deposition, and molecular features. Because the GI tract provides natural endoscopic access the technical feasibility of GI PAE has been investigated in small- and large-animal models, ex vivo human tissues, and a limited number of early human feasibility studies. Unlike single-scenario IVPA systems, GI applications must address diverse anatomical structures, including the intestinal tract, stomach, and esophagus, as well as multiple disease targets. These demands impose high requirements on probe configuration, imaging depth, FOV, scanning robustness, and multimodal compatibility.

Early GI PAE studies mainly used catheter-based probes to validate intraluminal microvascular imaging. Such probes are suitable for relatively narrow and tubular organs, such as the intestinal tract, where miniaturized optical-resolution designs can obtain high-resolution vascular information. Endoscope-channel-compatible PAE-EUS mini-probes further improved clinical usability by enabling imaging through standard endoscopic working channels [17]. Functional GI PAE based on optical heterodyne ultrasound detection enabled oxygenation-related assessment of rectal inflammation (Fig. 5**C**), while optical-resolution PAE was applied to rectal tumor evaluation through quantitative microvascular features such as vessel diameter, density, and tortuosity [18], [36]. These studies indicate that GI PAE has progressed from simple microvascular visualization toward functional disease assessment.

However, conventional catheter-based probes are less suitable for larger or irregular luminal organs, where stable contact, FOV coverage, and working-distance variation become major challenges. To address these issues, balloon-based PAE-EUS probes have been developed. The balloon structure improves probe-tissue coupling and helps stabilize imaging in deformable intestinal segments. By combining ultrasound-based wall-structure imaging with photoacoustic signals from hemoglobin and collagen, these systems have enabled assessment of intestinal strictures, Crohn's disease-related fibrosis, and inflammation [25], [26], [27]. Nevertheless, balloon probes are mainly suited for segmental evaluation of the intestinal wall and may be less convenient for broad screening or highly flexible navigation.

Capsule-based PAE provides another route for GI translation. Capsule PAE was developed to achieve circumferential esophageal vascular imaging and to improve compatibility with clinical esophageal screening workflows [16]. More recently, tethered PAE and OCT capsule endoscopy enabled label-free assessment of Barrett's esophageal neoplasia, combining PAE-derived vascular-functional information with OCT-derived mucosal microstructure [23]. Capsule systems are advantageous for large-FOV and circumferential imaging, especially in the esophagus, but their relatively large diameter, limited maneuverability, and system complexity still restrict broader use in smaller or tortuous luminal organs.

In parallel with probe evolution, the performance requirements of GI PAE have also shifted. Early studies mainly focused on vascular morphology, whereas recent work increasingly emphasizes functional, molecular, and multimodal assessment. Dual-wavelength functional PAE has been used to estimate blood oxygen saturation and quantify image-derived vascular features for experimental inflammation assessment [18]. Multiwavelength PAE–EUS systems have demonstrated relative hemoglobin- and collagen-associated contrast in experimental studies of intestinal inflammation and fibrosis [25], [26], [27]. Molecularly targeted PAE has been used for in vivo staging of early mucosal tumors, extending GI PAE from endogenous vascular contrast toward biomarker-oriented imaging [24], [94]. Multimodal systems integrating PAE with ultrasound (Fig. 5**D**), OCT, or optical microscopy further provide complementary information on mucosal microstructure, vascular function, and molecular contrast [23], [47], [95], [96]. Recent advances, including high-fluence PAE-EUS, GI PAE, dual-scanning PAEM, all-fiber PAEM for oxygenation-related disease mechanisms, ultrafast decoupled coaxial core perfusion imaging, and translational PAE-EUS catheters for pig GI inflammation and fibrosis models, further support the movement of GI PAE toward functional and endoscope-compatible and workflow-oriented platforms [22], [35], [43], [85], [97], [98].

Overall, GI PAE probes have evolved from catheter-based to capsule-based designs and from single-modality microvascular imaging toward multimodal functional and molecular evaluation. This trajectory shows that GI PAE has expanded beyond basic vascular visualization toward experimental functional, compositional, and multimodal assessment. Future development should focus on endoscope-compatible probe design, motion correction, standardized quantification of related biomarkers, and larger-scale human validation. These advances will be essential for moving GI PAE from promising experimental imaging toward clinically actionable diagnosis and decision support.

### 3.3 Emerging applications: reproductive, urinary, respiratory, and surgical guidance

With the progress of PAE in cardiovascular and GI imaging, its value in functional assessment and disease characterization has promoted its extension to other luminal and cavity organs, including the reproductive tract, urinary system, respiratory, and surgical fields. These scenarios differ substantially in anatomical access, target information, probe configuration, and workflow requirements, demonstrating that PAE is not only an organ-specific imaging tool but also a scenario-adaptable platform for intraluminal, intracavitary, or minimally invasive functional imaging. Table 3 selected studies prioritize the information obtained and demonstrate application-relevant information such as vascular morphology, oxygenation, lipid composition, fibrosis, molecular contrast, or surgical guidance, ranging from unimodal to multimodal studies.

**Table 3:** Representative PAE applications in different clinical scenarios. PAE, photoacoustic endoscopy; IVPA, intravascular photoacoustic imaging; IVUS, intravascular ultrasound; EUS, endoscopic ultrasound; GI, gastrointestinal; OCT, optical coherence tomography; PA, photoacoustic; COPD, Chronic obstructive pulmonary disease; 3D, three dimensional; IHR-NPs, ICG-HSA-RGDfk nanoparticles; N.A, not applicable.

**Application scenario**	**Model / object**	**Target organ or disease**	**Evidence level**	**Imaging configuration**	**Acquired information**	**Probe / device size**	**Year, team** **and ref.**
Cardiovascular System	Rabbit	Atherosclerotic plaque	Ex vivo	IVUS-guided multi-wavelength (1200–1230 nm) IVPA imaging	Lipid-specific plaque contrast and vascular wall structure	Only IVUS catheter	2010, [37] S.Y. Emelianov
	Human	Coronary atherosclerotic plaque	Ex vivo	Dual-wavelength (1718 and 1734 nm) IVPA - IVUS	Distinguish lipids in plaques or in peri-adventitial tissue	*N.A.*	2015, [32] G.v.Soest
	Rabbit	Atherosclerotic plaque	In vivo	IVPA -IVUS- Molecular probe	Molecular imaging of angiogenesis with IHR-NPs	0.9 mm OD	2021, [87] F. Cao
	Rabbit	Atherosclerotic plaque	In vivo	High-speed IVPA-IVUS	Lipid distribution and lumen / vessel-wall contour	0.9 mm OD	2020, [76] X. Gong
	Rabbit	Abdominal aorta with plaque	In vivo	Quad-modality IVPA-IVUS-OCT-photoacoustic elastography	Lipid, morphology, fine structure and elasticity	0.97 mm OD	2023, [88] S. Yang
Digestive system	Rat	Rectum	Ex vivo	Single-modality PAE	Vascular network	4.2 mm OD	2009, [14] L.V. Wang
	Mouse	Pancreatic tumor	In vivo	Dual-modality PAE-FI	Vascular and tumor tissue information	1 mm OD	2017, [94] H. Jiang
	Rabbit	Intestine with inflammation-fibrosis	In vivo	PAE-EUS balloon, multi-wavelength	Hemoglobin, collagen deposition and wall structure	7 mm OD (balloon/capsule)	2019–2025, [25], [26], [27] G. Xu
	Rat	Rectum with acute inflammation	In vivo	Dual-wavelength PAE (532 and 558 nm)	Vascular density and blood oxygen saturation	2.0 mm OD	2022, [18] B.-O. Guan
	Rat	Rectum with primary tumor	In vivo	Single-modality PAE	Vessel diameter and tortuosity	2.5 mm OD (with sheath)	2024, [36] X. Gong
	Human	Human Barrett’s esophageal neoplasia specimens	Ex vivo human- tissue validation	Dual-modality PAE-OCT	Mucosal microstructure and vascular-functional information	12.5 mm OD (capsule)	2026, [23] V. Ntziachristos
	Pig/ human-oriented	GI inflammation and fibrosis	In vivo	Translational PAE-EUS catheter	Inflammation, fibrosis and wall structure	Endoscope-compatible	2026, [98] G. Xu
Reproductive system	Human	Cervical remodeling / microvasculature	In vivo	Transvaginal OR-PAE / PA spectroscopy	Cervical microvasculature and connective-tissue remodeling	∼20 mm probe / transvaginal access	2018, [15] L.V. Wang
	Rat	Endometrial microvessels	In vivo	Single-modality PAE	3D endometrial microvasculature	1.5 mm OD (with sheath)	2024, [19] X. Gong
	Porcine/human	Ovary	Ex vivo	Tri-modality PAE-EUS-OCT	Vasculature, ovarian morphology and structure	5 mm OD	2011, [99] Q. Zhu
Urinary system	Rabbit	Urethra and bladder	In vivo	Dual-modality PAE-EUS	3D microvascular network and urinary morphology	2.8 mm OD	2022, [103] J.-M. Yang
Respiratory system	Rabbit	Airway with COPD	In vivo	Dual-modality PAE-EUS	Tracheal microvessel diameter/density and mucosal thickness	2.1 mm OD	2025, [105] J. Zhang
Surgical guidance	Human cadaver	Hysterectomy	Cadaveric study	Dual-wavelength (690 and 750 nm)	Ureter-artery differentiation and distance feedback	*N.A.*	2021, [107] M.A.L. Bell
Multi-scenario	Human, rabbit, rat	Oral mucosa, cervix, rectum	In vivo	Single-modality PAE	Microvascular parameters and blood oxygenation	5.5 mm OD	2025, [20] L. Xi

In the reproductive system, PAE has shown potential for early translational applications. Early work integrated OCT, ultrasound, and PAE for ex vivo characterization of porcine and human ovarian tissues, combining superficial microstructure, deep morphology, and vascular contrast, demonstrating potential for ovarian cancer detection and diagnosis [99]. Subsequent studies extended PAE to the cervix and uterus. For pregnancy-related cervical changes, infrared photoacoustic spectroscopy was used to assess cervical connective-tissue remodeling, while transvaginal fast-scanning optical-resolution PAE (OR-PAE) enabled human cervical microvascular imaging, supporting studies of cervical remodeling mechanisms and preterm-birth risk [15], [100]. In uterine imaging, intrauterine PAE-EUS imaging confirmed the feasibility of ex vivo uterine-wall perfusion visualization [48], while recent intrauterine PAE enabled in vivo three-dimensional visualization and quantitative analysis of rat endometrial microvessels (Fig. 5**E**) [19], providing preclinical evidence that intrauterine PAE can visualize and quantify endometrial microvascular features in vivo.

In the urinary system, several studies have demonstrated the feasibility of urinary PAE through natural transurethral access. An early urogenital PAE was developed for potential human urogenital imaging, using a rigid side-scanning probe to target diseases such as endometrial and prostate cancers [101]. Transurethral PAE was subsequently proposed for prostate cancer localization and therapy monitoring in simulation studies [102]. More recently, a tapered catheter-based transurethral PAE-EUS probe enabled in vivo imaging of the rabbit urethra and bladder, providing both urinary-wall morphology and microvascular information (Fig. 5**F**) [103]. In addition, miniaturized all-optical PAE using MEMS mirror scanning and MRR ultrasound detection has demonstrated three-dimensional microvascular imaging of canine bladder tissue, providing an early endoscopy-oriented prototype for future bladder PAE [104]. These studies suggest that urinary PAE has potential for minimally invasive assessment of the urethra, bladder, and prostate.

Respiratory applications further demonstrate the functional value of PAE. A recent acoustic-optic confocal PAE-EUS tracheal system enabled in vivo assessment of tracheal microvessel diameter, vascular density, and mucosal-layer thickness in chronic obstructive pulmonary disease models (Fig. 5**G**) [105]. More recently, intra-tracheal PAE was applied to track inhaled nano-adjuvant transport across tracheal layers in vivo, expanding respiratory PAE from airway-structure and vascular assessment to dynamic monitoring of inhaled drug-delivery processes [106].

PAE has also shown promise in additional scenarios. Multi-scenario PAE has demonstrated functional imaging in human oral mucosa, rabbit cervix, and rat rectum by adapting imaging geometry to different anatomical environments (Fig. 5**H**) [20]. For surgical guidance, photoacoustic imaging was used to distinguish the ureter from the uterine artery during hysterectomy in human cadaver studies with dual-wavelength contrast, demonstrating the feasibility of real-time ureter–artery differentiation and distance estimation during simulated surgical procedures [107].

Overall, these emerging applications broaden the clinical boundary of PAE. Future development should focus on scenario-adaptive probe design, multimodal integration, quantitative validation, scanning robustness, safety assessment, and workflow-compatible engineering, providing an engineering basis for future clinical evaluation of PAE in diagnosis and image-guided intervention.

## 4 Clinical translational challenges and future perspectives

**Fig. 6:**
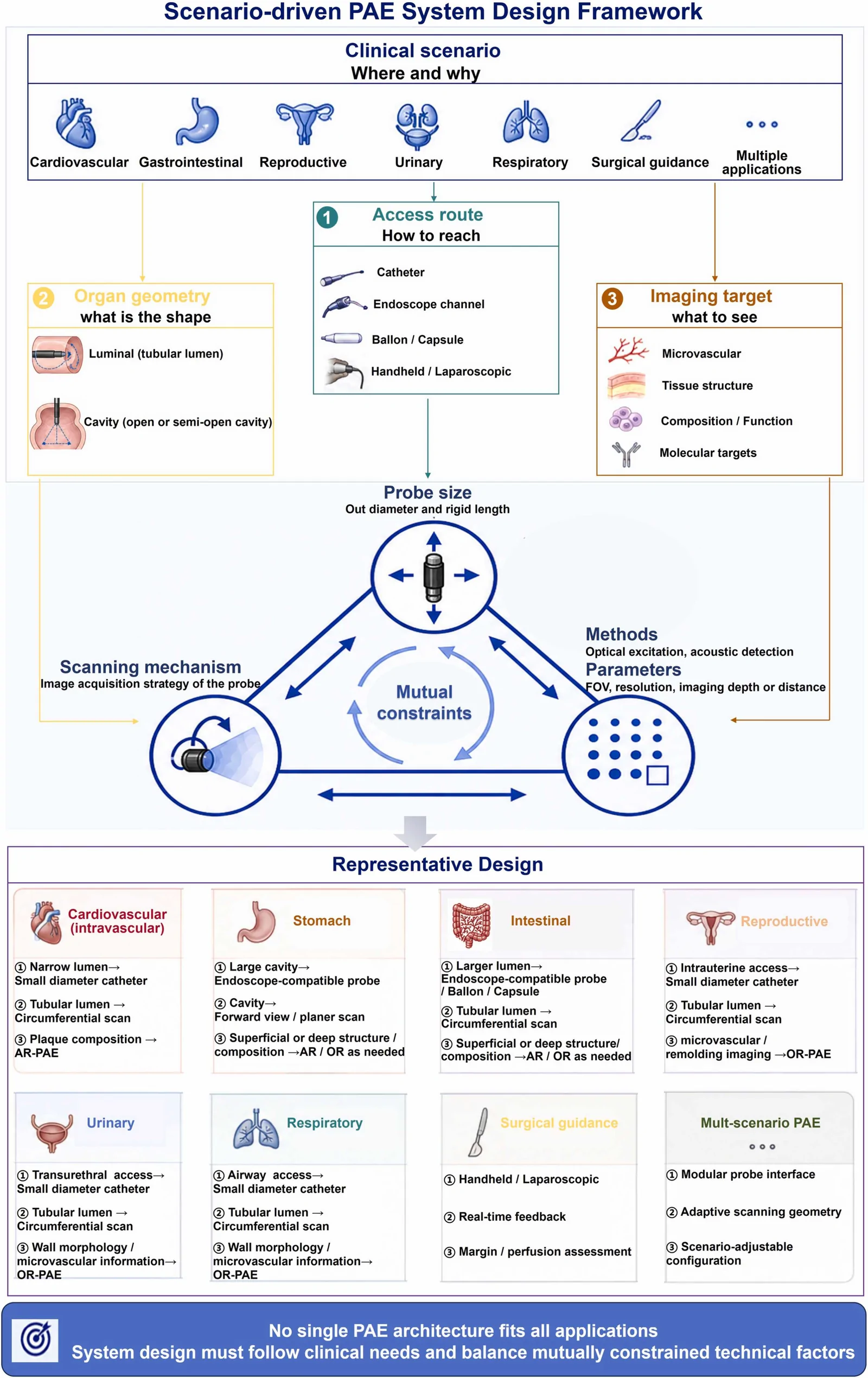
Scenario-driven PAE system design framework. Clinical scenarios, defined by the target organ, tissue, and application, determine the access route, organ geometry, and imaging target. These factors further guide the selection of probe size, scanning mechanism, imaging methods, and key performance parameters, including optical excitation, acoustic detection, FOV, resolution, and imaging depth or working distance. Representative design outputs are summarized for cardiovascular, gastrointestinal, reproductive, urinary, respiratory, surgical-guidance, and multi-scenario PAE applications. The framework highlights that no single PAE architecture is suitable for all applications; instead, system design should follow clinical needs and balance mutually constrained technical factors. PAE, photoacoustic endoscopy; FOV, field of view; AR, acoustic resolution; OR, optical resolution. Notes: Some illustrative elements in this figure were generated using ChatGPT and were further arranged and edited by the authors.

PAE has progressed from early proof-of-concept systems to increasingly application-specific preclinical platforms. Nevertheless, clinical evidence remains limited, with most studies involving phantoms, animal models, ex vivo human tissues, or small-scale feasibility investigations. Future systems should be designed according to specific clinical scenarios rather than by maximizing individual technical metrics. As summarized in Fig. 6, each clinical scenario determines the access route, organ geometry, and imaging target, which in turn guide probe size, scanning mechanism, imaging methods, and key performance parameters under mutual engineering constraints.

For example, cardiovascular imaging requires small-diameter, high-speed, lipid-sensitive catheters; GI imaging requires large-FOV, motion-robust, endoscope-compatible platforms; reproductive and urinary applications require safe access and miniaturized probes; and airway or surgical-guidance applications require real-time feedback and operational stability.

However, scenario-specific system design alone is insufficient for clinical translation. Laser safety should also be reported quantitatively. Per-pulse radiant exposure, repetition rate, total exposure time, and cumulative exposure should be compared with wavelength- and exposure-duration-dependent maximum permissible exposure (MPE) limits defined by laser-safety standards such as ANSI Z136.1 or IEC 60825–1. In transcutaneous nanosecond-pulsed photoacoustic imaging, conservative practical fluence benchmarks are often taken as approximately 20 mJ/cm² in the visible range and approximately 100 mJ/cm² near 1064 nm. However, these values should be regarded as engineering reference levels for transcutaneous exposure rather than established safety limits for luminal mucosa, vessel walls, or blood-filled intravascular environments. The exact MPE must be calculated according to wavelength, pulse duration, exposure duration, spot size, repetition rate, and multiple-pulse correction factors. Early IVPA safety studies evaluated laser-induced cellular and vascular damage at lipid-relevant wavelengths. Sowers et al. showed that high single-pulse fluence was unlikely to be the dominant cause of cell death; instead, cumulative exposure and heat accumulation were more relevant safety constraints [92]. Subsequent in vivo swine carotid studies further showed that vascular medial necrosis could occur above specific cumulative exposure levels, with a reported damage threshold of 50 J/cm² at 1720 nm [93], whereas previously reported IVPA lipid-imaging protocols operated below this threshold. These studies indicate that safety assessment for PAE should not rely only on nominal MPE comparison. For intravascular and intracavitary PAE, future studies should report delivered pulse energy, estimated tissue-surface fluence, repetition rate, cumulative exposure, catheter dwell time, tissue-contact condition, optical coupling environment, local temperature change, and histological or cellular safety assessment.

For transcutaneous nanosecond-pulsed photoacoustic imaging, conservative fluence benchmarks commonly used in engineering practice are approximately 20 mJ/cm² in the visible range and approximately 100 mJ/cm² near 1064 nm. These values, however, should be regarded only as practical reference levels rather than universal safety limits. The exact MPE must be calculated according to ANSI Z136.1 or IEC 60825–1, taking into account the excitation wavelength, pulse duration, total exposure time, beam diameter or spot size, repetition rate, and multiple-pulse correction factors. For intravascular and intracavitary PAE, MPE comparison should be supplemented by tissue-contact information, catheter dwell time, temperature monitoring, and histological or cellular safety assessment.

In parallel, clinical translation will require workflow-compatible engineering, including sterilizable or disposable probes, catheter robustness, motion correction, and integration with existing endoscopic platforms. Multimodal integration and AI-assisted analysis may further support clinical interpretation, but their value must be validated in standardized and prospective studies.

## 5 Conclusions and outlook

PAE has developed from early side-viewing prototypes into a diverse family of catheter-, capsule-, array-, and fiber-based imaging systems for intraluminal and intracavitary applications. These platforms have demonstrated vascular, structural, functional, and compositional imaging in phantoms, animal models, ex vivo human tissues, and a limited number of early human feasibility studies. However, the available evidence remains heterogeneous, and routine clinical utility has not yet been established for most proposed indications. Future development should prioritize scenario-specific system design, end-to-end calibration, measurement repeatability and reproducibility, motion correction, safety, manufacturability, sterilization, and compatibility with established endoscopic workflows. Directly observed PA signals and image-derived morphological features should be distinguished from model-derived estimates, such as blood oxygen saturation and relative lipid- or collagen-associated contrast. Prospective studies comparing PAE with standard-of-care imaging are therefore required to determine whether it provides incremental value in disease detection, risk stratification, treatment guidance, or therapeutic monitoring. The next phase of PAE development is not simply to improve individual performance metrics, but to translate technically robust measurements into reproducible and clinically meaningful evidence.

## CRediT authorship contribution statement

**Qingrong Xia:** Writing – original draft, Conceptualization. **Xiaojing Gong:** Writing – review & editing.

## Declaration of Generative AI and AI-assisted technologies in the writing process

In this study, ChatGPT was used for embellish elements of illustration in Figs. 1 and 6. All AI-assisted outputs were reviewed and verified by the authors.

## Declaration of Competing Interest

The authors declare that they have no known competing financial interests or personal relationships that could have appeared to influence the work reported in this paper.

## Data Availability

Data will be made available on request.
